# New UN envoy joins campaign to end TB

**DOI:** 10.2471/BLT.15.030315

**Published:** 2015-03-01

## Abstract

Eric Goosby tells Fiona Fleck why tuberculosis prevention and care should be integrated into a comprehensive package of health services for which universal health coverage is essential.

Q: What are the key challenges of the global tuberculosis response?

A: We have known about tuberculosis for hundreds of years and for the past 50 years we have known how to prevent, diagnose and, in most instances, cure it. The major challenge – in rich and poor countries alike – is that tuberculosis has always been more prevalent among people in lower socioeconomic groups, who often have difficulty accessing treatment and completing the full course of treatment as prescribed (an average treatment course is six months for drug-susceptible tuberculosis and up to two years for drug-resistant types of the disease). Other challenges include the need to improve diagnostics for tuberculosis, for more prompt detection, and to develop medicines that would allow for shorter courses of treatment. In Africa, a major challenge is, of course, HIV-associated tuberculosis, while drug-resistant forms of the disease, especially in eastern Europe and Central Asia, require focused efforts in the global response.

Q: How do you plan to address these challenges?

A: The World Health Organization’s (WHO) post-2015 End TB Strategy sets a clear response. In my position as Special Envoy on Tuberculosis and in partnership with WHO, I will endeavour to work with ministries of health – particularly those in high-burden countries – to strengthen prevention efforts through aggressive contact tracing, so that people with tuberculosis are identified as early as possible and provided with treatment. If we can get patients on treatment, we can cure them. I am confident that by refocusing our attention on the basics, we can strengthen service delivery in most countries. Also, by providing technical assistance to help boost the capacity of each ministry and by drawing on community-based strategies, we can save many lives. I will also work to build links with other ministries such as those responsible for social welfare, justice and immigration to address the social determinants of tuberculosis. In addition, I will also work with the academic and private sectors to advocate for more research and development of new vaccines, drugs and diagnostics.

“If we can get patients on treatment, we can cure them.”

Q: How will you support the new WHO End TB Strategy?

A: First, my office will work with WHO and its partners to encourage countries to adopt the strategy with its commitment to drive down deaths globally by 95% and end the global epidemic by 2035. I will support WHO to continue to provide countries with the relevant guidelines and norms they need for their tuberculosis programmes, while raising the global profile of tuberculosis on the political and development agenda. Second, we will support strategies to target clinical officers, nurses and other health workers, who play a huge role in the diagnosis and treatment of patients to boost their skills and understanding of the new strategy. Finally, ensuring the engagement of civil society and the private sector will also be crucial for the achievement of the ambitious targets of the End TB Strategy. Therefore, we will focus on engaging the community – both infected and affected – to raise awareness of the need to identify people at risk of tuberculosis, to diagnose those infected and to retain them in care for prophylaxis or treatment. In this way, we will empower people affected by tuberculosis to demand services from their public health systems. 

Q: Can you describe your early experience treating patients with HIV? What was remarkable about this experience and what lessons do you bring from this to the global fight against tuberculosis?

A: I trained at San Francisco General Hospital (SFGH) from 1974 to 1984. Many of the first patients with HIV came into our emergency department and – in the early years – filled inpatient units around the city. Our centre initially saw more HIV cases than anywhere else in the United States and San Francisco General – part of the University of California Clinics and Hospitals – was the first hospital to devote whole units to HIV: both an outpatient clinic, called Ward 86, and an inpatient unit called Ward 5. Since 1982, SFGH has continued to deliver care to HIV-infected people, their families and friends. In the early 1980s the epidemic just bubbled up in our community and, at first, we did not know what it was or how it spread, so people were afraid of becoming infected and bringing it home to their families. It wasn’t until 1984 that the human immunodeficiency virus (HIV) was identified and diagnostic test for HIV developed. 

There are parallels between HIV infection and tuberculosis, especially with regard to the stigma that has grown up around these two diseases. People are afraid of becoming infected and, as a result, the responses of individuals, institutions, schools and workplaces to limit exposure are understandable, but often not based on science. The stigma that has built up around tuberculosis slowly over hundreds of years still remains a major barrier to access for, and retention of, patients in treatment.

Q: You say that while stigma is not based on science, it is understandable. How then can we fight this often overwhelming barrier to tackling tuberculosis?

A: For diseases such as HIV infection and tuberculosis, that require treatment over months or longer and regular contact with the medical system, we need to understand how stigmas arise and present a barrier to successful treatment. We need to have strategies in place to counter them, both educational as well as structural ones, strategies that allow people to access treatment without standing out. That may mean, for example, receiving treatment outside working hours or providing methods for people to take their medication home. 

Q: One of the key fronts on which the public health community is fighting tuberculosis is in trying to improve access to treatment, how will you support these efforts in your new role?

A: Anti-mycobacterial medicines were first developed in the 1940s and only two new drugs for tuberculosis have emerged in the past 40 years. The pipeline for new tuberculosis drug development has been feeble compared with that for HIV infection and other chronic diseases. It is high time that we addressed this problem. It is not acceptable that every day in 2015 about 4000 people will die of this curable disease. We need more new drugs and drug combinations to treat tuberculosis. That is why I will be seeking engagement and dialogue with key private sector stakeholders.

Q: When you were in charge of HIV Services at the US Public Health Service in the early 1990s, you reported the lack of access to health care that led to poorer outcomes among African-Americans with HIV infection. How do you rate the success of the Affordable Care Act in the United States, which is in line with the global drive towards universal health coverage?

A: It’s a wonderful moment in the public health narrative. The Affordable Care Act in the United States is part of the global momentum towards universal health coverage and has succeeded in bringing most of the 15% of Americans, who did not have health insurance – many of them from low socioeconomic groups or unemployed – into a health insurance relationship that covers their basic health needs.

Q: How do you see the role of universal health care in the global fight against tuberculosis?

A: It’s the first time that the United Nations Member States including the donor countries have seriously accepted the goal of universal health coverage. The public health community is finally talking about a comprehensive package of services and not just dividing up diseases with a delivery system that creates redundancies. Such delivery systems waste money and are neither efficient nor effective for the hospitals and clinics that provide the services, or convenient for patients seeking these services. Often, because of the way we fund public health programmes in resource-constrained settings, people must go to separate sites or come to health centres on different days for different needs. There are separate clinics for family planning and maternal and child care. Immunizations are sometimes integrated into primary care and sometimes done on a separate site. Tuberculosis and HIV services are often provided by specialized clinics. We need to create comprehensive integrated service sites, where individuals receive a comprehensive assessment of their health needs and receive all possible health services in – as far as possible – one place and at one time.

Q: You led the global response to HIV at PEPFAR, which has been massively criticized over certain aspects of its approach, including doing too little to address the double burden of HIV infection and tuberculosis. What do you say to critics of the programme?

A: The US President's Emergency Plan for AIDS Relief (PEPFAR) was a remarkable programme started in 2003 by President [George W] Bush. When the Obama administration took it over in 2009, PEPFAR had already set up delivery systems, often from the ground up, and provided comprehensive care for HIV-positive individuals in the 15 most heavily affected countries. By the time I left in 2013, we had a significant footprint in 73 countries. HIV infection is the leading motor for the rise in tuberculosis. In 2009, 1.7 million people were being treated by PEPFAR for HIV infection, a third of them had tuberculosis as well. By 2013, the total number of patients on treatment had expanded to 7.3 million, this is with an 11% decrease in funding. The PEPFAR programme had the largest expansion of tuberculosis services for people living with HIV on the planet in that same time period. President Bush started PEPFAR, but President Obama took it to scale expanding the number of people diagnosed and treated for tuberculosis and HIV by seven or eight times. I am very proud to have been part of that expansion.

